# Use of Rapid, Point-of-Care Assays by Private Practitioners in Chennai, India: Priorities for Tuberculosis Diagnostic Testing

**DOI:** 10.1371/journal.pone.0155775

**Published:** 2016-06-15

**Authors:** Liza Bronner Murrison, Ramya Ananthakrishnan, Sumanya Sukumar, Sheela Augustine, Nalini Krishnan, Madhukar Pai, David W. Dowdy

**Affiliations:** 1 Department of Epidemiology, Johns Hopkins Bloomberg School of Public Health, Baltimore, Maryland, United States of America; 2 Center for Tuberculosis Research, Johns Hopkins University, Baltimore, Maryland, United States of America; 3 REACH, Chennai, India; 4 McGill International TB Centre & Department of Epidemiology, Biostatistics, and Occupational Health, McGill University, Montreal, Quebec, Canada; Cambridge University, UNITED KINGDOM

## Abstract

**Setting:**

Private practitioners are frequently the first point of healthcare contact for patients with tuberculosis (TB) in India. As new molecular tests are developed for point-of-care (POC) diagnosis of TB, it is imperative to understand these individuals’ practices and preferences for POC testing.

**Objective:**

To evaluate rapid testing practices and identify priorities for novel POC TB tests among private practitioners in Chennai.

**Design:**

We conducted a cross-sectional survey of 228 practitioners practicing in the private sector from January 2014 to February 2015 who saw at least one TB patient in the previous year. Practitioners were randomly selected from both the general community and a list of practitioners who referred patients to a public-private mix program for TB treatment. We used standardized questionnaires to collect data on current practices related to point-of-care diagnosis and interest in hypothetical POC tests. We used multivariable Poisson regression with robust estimates of standard error to calculate measures of association.

**Results:**

Among 228 private practitioners, about half (48%) utilized any rapid testing in their current practice, most commonly for glucose (43%), pregnancy (21%), and malaria (5%). Providers using POC tests were more likely to work in hospitals (56% vs. 43%, P = 0.05) and less likely to be chest specialists (21% vs. 54%, P<0.001). Only half (51%) of providers would use a hypothetical POC test for TB that was accurate, equipment-free, and took 20 minutes to complete. Chest specialists were half as likely to express interest in performing the hypothetical POC TB test in-house as other practitioners (aPR 0.5, 95%CI: 0.2–0.9). Key challenges to performing POC testing for TB in this study included time constraints, easy access to local private labs and lack of an attached lab facility.

**Conclusion:**

As novel POC tests for TB are developed and scaled up, attention must be paid to integrating these diagnostics into healthcare providers’ routine practice and addressing barriers for POC testing.

## Introduction

Despite efforts to increase case detection, 3 million new tuberculosis (TB) cases are not notified to national TB programs each year; over one-quarter (27%) of these “missed cases” are estimated to occur in India [1,2]. Improvements in case detection through increased diagnostic capabilities could substantially reduce the gap between notified cases and estimated incidence [1]. In particular, a rapid, low cost, accurate diagnostic assay for TB that can be used at the point of care to make rapid treatment decisions would be a major advance in efforts to reach these individuals [3]. Target product profiles for such point-of-care (POC) tests have been developed and published [4].

The Government of India’s Revised National TB Control Program (RNTCP) provides free TB healthcare services; however, up to 85% of patients experiencing TB-related symptoms in urban Indian settings first seek healthcare in the private sector [5–7]. Thus, any efforts to implement novel TB diagnostic tests in India (and other similar countries) would need to engage private practitioners. As TB control programs worldwide begin to prioritize molecular diagnostics that are more sensitive than smear microscopy [8], it is imperative to consider the implementation of tests for TB that could be deployed at the point-of-care [9,10].

A critical element of implementing any novel diagnostic test is to evaluate existing capacity for, and acceptability of, the test among those healthcare providers who would be responsible for using it [3,10]. For example, if primary care providers are already using rapid tests for diseases such as malaria and HIV, then they may be more likely to adopt a novel POC TB test in future. In this context, we sought to evaluate point-of-care testing practices and to identify priorities for novel POC tests for TB among private practitioners in Chennai, India.

## Methods

### Setting

Chennai is the sixth largest city in India with a population of 4.6 million [11]. The estimated prevalence of bacteriologically confirmed pulmonary TB was approximately 349 cases per 100,000 population in Chennai in 2012 (versus 211 per 100,000 nationwide in 2013) [1,12]. We collaborated with the Resource Group for Education and Advocacy for Community Health (REACH) to perform cross-sectional interviews with urban private medical practitioners (PPs) in Chennai. As a non-governmental public-private mix (PPM) organization, REACH collaborates with Corporation of Chennai to involve PPs in the RNTCP [13]. Through advocacy and outreach efforts, REACH networks with PPs in Chennai that refer patients with TB to REACH’s four PPM Centers located in private hospitals. Patients referred to REACH receive free TB treatment with supervision by REACH staff or community DOT (Direct Observed Therapy) providers.

### Sample selection and data collection

From January 2014 through February 2015, we recruited and enrolled qualified, allopathic medical practitioners of all specialties working in the private sector in Chennai. REACH study staff used a structured movement algorithm to recruit a maximum of five PPs per health facility and minimum of five PPs in each of Chennai’s ten 2011 census tract zones. We also randomly recruited PPs from REACH’s database of PPM-referring PPs. All consenting, qualified PPs with formal medical training who diagnosed at least one patient with pulmonary TB (PTB) in the year prior to recruitment were eligible for interview. Up to three attempts were made to complete the interview after a PP agreed to participate. The structured interviews, performed privately in the PPs’ offices by trained study staff, lasted 15 to 20 minutes and included PP socio-demographic information (with the exception of age, which was deemed to be potentially identifiable during ethical review), self-reported education (medical doctor/MD, master of surgery/MS, or bachelor of medicine and surgery/MBBS degrees), patient volume, clinical specialty, TB disease knowledge, and diagnostic and treatment practices. Interviewers asked practitioners about POC testing practices for pregnancy, glucose, HIV, TB antibody, malaria, syphilis, hepatitis, dengue, and typhoid using reference cards listing the tests. All POC tests were described as diagnostic tests used to make quick management decisions in the same visit (i.e. while the patient waits) in the clinic or practice, including those performed in any attached lab facilities.

### Priorities for a novel point-of-care test

As part of the interview, PPs were asked about a hypothetical POC test for TB “*that could be used to replace the tests that [they] currently use*. *This test could potentially be done rapidly in [their] clinic*, *like a pregnancy test or blood glucose test*,” and was described as accurate, taking 20 minutes to perform, and requiring no equipment. PPs were asked if they would perform this test in their clinic (versus sending patients out to a lab or not performing it), and about the maximum amount they would be willing to pay to use this POC test for most patients with symptoms of TB.

### Statistical analysis

We assessed univariate differences in proportion using Pearson’s (or Fisher’s exact) test. We evaluated differences in medians for continuous variables using the non-parametric Wilcoxon test. To estimate univariate and multivariable associations with self-reported preference to use a new POC test for TB in-house, we used Poisson regression models with robust standard errors to calculate prevalence ratios, as the outcome was not rare and log-binomial models failed to converge [14]. We examined collinearity using variance inflation factors, which were below 1.5 for all covariates included in the model.

### Ethical considerations

This study protocol was approved by the Johns Hopkins Bloomberg School of Public Health Institutional Review Board, McGill University Health Center Biomedical Research Ethics Review Board, and REACH Independent Ethics Committee. Written informed consent was obtained from all practitioners prior to being interviewed for the study.

## Results

### Description of population and point-of-care testing practices

Among 249 eligible PPs recruited, 228 (92%) completed the interview. Of these, 161 (71%) were randomly selected within Chennai city and 67 (29%) were randomly selected from REACH’s PPM database. More than half (56%) of PPs worked in private standalone clinics, and 17% were chest specialist physicians (Table 1). The median number of TB patients seen per year was 12 [IQR 4–28]: 65 [IQR 20–125] among chest specialists versus 8 [IQR 4–20] among other practitioners (P<0.001). Just under half (110/228, 48%) of all PPs utilized any POC tests in their clinics; these included glucose (43%), pregnancy (21%), malaria (5%), hepatitis (4%), dengue (4%), typhoid (4%), and HIV (2%) POC tests. Providers who used POC tests were more likely to be women, work in a private hospital, have fewer years of practice experience, and be medical doctors (Table 1). Among practitioners who used point-of-care testing, the median volume was 38 POC tests [IQR 15–100] per month, median turnaround time was 5 minutes [IQR 3–5], and median cost to patients was 50 INR [IQR 50–88] per test (approximately US$0.79) (Table 2). Support staff or nurses performed 55% (60/110) of POC tests, while practitioners interpreted the majority (98/110, 89%) of test results. Only one-quarter (27/110, 24%) of PPs kept records of the POC test results.

**Table 1:** Study population characteristics among private practitioners comparing point-of-care (POC) testing practices in Chennai, India (n = 228).

		Point-of-Care Testing Practices		
Characteristic	Total (n = 228) n(%)	Any POC Testing* (n = 110) n(%)	No POC Testing (n = 118) n(%)	P**
**Gender**				
Male	160 (70)	68 (62)	92 (78)	**<0.01**
Female	68 (30)	42 (38)	26 (22)	
**Education level**				
MBBS	80 (35)	37 (34)	43 (36)	0.85
MS	11(5)	6 (5)	5 (4)	
MD	137 (60)	67 (61)	70 (59)	
**Type of facility**				
Government with private practice in evening	22 (10)	8 (7)	14 (12)	**0.02**
Private standalone clinic or polyclinic	129 (56)	55 (50)	74 (63)	
Private hospital or nursing home	77 (34)	47 (43)	30 (25)	
**Years practicing**				
Median [IQR]	20 [15–30]	20 [14–28]	25 [15–30]	**0.02**
**Practitioner specialty**				
General medicine	153 (67)	77 (70)	76 (65)	**<0.001**
Chest/Pulmonary specialist	39 (17)	8 (7)	31 (26)	
Other^‡^	36 (16)	25 (23)	11 (9)	
**Number of patients diagnosed with TB annually**				
≤12 patients with TB	110 (48)	53 (48)	57 (48)	0.99
>12 patients with TB	118 (52)	57 (52)	61 (52)	
**Action for pulmonary TB diagnosis**				
Refer to RNTCP or PPM DOTS center	97 (42)	48 (44)	49 (42)	0.75
Treatment in private sector	131 (58)	62 (56)	69 (59)	
**Usage of sputum smear microscopy for TB**				
Orders at first patient visit for TB diagnosis	115 (50)	54 (49)	61 (52)	0.69
Not ordered at first patient visit	113 (50)	56 (51)	57 (48)	
**Knowledge of serological antibody test ban**				
Yes	126 (55)	60 (55)	66 (56)	0.83
No	102 (45)	50 (46)	52 (44)	
**Source of information on TB**^**†**^				
No sources/non-chest specialty	39 (17)	14 (13)	25 (21)	0.09
Journals, books, newspaper, newsletters	82 (36)	42 (38)	40 (34)	0.50
Internet	71 (31)	38 (35)	33 (28)	0.28
CME or workshop	99 (43)	46 (42)	53 (45)	0.64
Medical representative or colleague	25 (11)	11 (10)	14 (12)	0.65

MD, Medical doctor degree; MS, Master of surgery degree; MBBS, Bachelor of medicine and bachelor of surgery undergraduate degrees; RNTCP, Revised National TB Control Program; PPM DOTS, Public-Private Mix directly observed therapy short course.

*POC tests utilized included glucose (n = 97), pregnancy (n = 48), malaria (n = 12), hepatitis (n = 9), dengue (n = 9), typhoid (n = 9), and HIV (n = 4).

**Pearson's chi-squared (or Fisher's exact) test for categorical variables comparing practitioners that perform point-of-care (POC) diagnostic tests in-house versus ordering from a private lab; Wilcoxon test for continuous variables.

‡Other MD and MS practitioner specialties included Obstetrics and gynecology (n = 15), Pediatrician (n = 5), Surgeon (General/Orthopedic/Ophthalmologic) (n = 4), Diabetes Specialist (n = 6), Ear nose and throat (n = 2), Oncologist (n = 1), and Radiologist (n = 1).

†Categories are not mutually exclusive.

**Table 2:** Point-of-care testing practices and preferences among private practitioners currently using any POC tests by patient volume in Chennai.

		Point-of-Care Testing Preferences		
Characteristic	Total (n = 110) n(%)	≤12 Patients (n = 53) n(%)	>12 Patients (n = 57) n(%)	P*
**Practitioner level of training**				
MBBS/MS/MD (Non-chest specialty)	102 (93)	52 (98)	50 (88)	0.06
MD (Chest/Pulmonary specialty)	8 (7)	1 (2)	7 (12)	
**Type of facility**				
Private hospital or nursing home	55 (50)	20 (38)	35 (61)	**0.02**
Private standalone clinic or polyclinic*	55 (50)	33 (62)	22 (39)	
**Number of tests per month**				
Median [IQR]	38 [15–100]	20 [15–55]	75 [30–120]	**<0.001**
**Time to get test results (minutes)**				
Median [IQR]	5 [3–5]	5 [4–5]	5 [3–5]	0.30
**Staff performing test**				
Practitioner/Doctor	50 (45)	29 (55)	21 (37)	0.06
Support Staff/Nurse	60 (55)	24 (45)	36 (63)	
**Staff interpreting test results**				
Practitioner/Doctor	98 (89)	45 (85)	53 (93)	0.23
Support Staff/Nurse	12 (11)	8 (15)	4 (7)	
**Cost to patients per test**				
Median [IQR], (INR)	50 [50–88]	50 [50–100]	50 [50–75]	0.12
Median [IQR], (USD,$)	0.73 [0.73–1.28]	0.73 [0.73–1.45]	0.73 [0.73–1.09]	
**Keeps record of test results**				
Yes	27 (25)	31 (58)	52 (91)	**<0.001**
No	83 (75)	22 (42)	5 (9)	
Reasons for performing POC tests**				
Results available immediately	96 (87)	42 (79)	54 (95)	0.14
Convenience for patients	70 (64)	30 (57)	40 (70)	0.46
Diagnosis and treatment in same visit	35 (32)	19 (36)	16 (28)	**0.02**
More affordable for patients	13 (12)	5 (10)	8 (14)	0.38
**Interested in using novel POC TB test**				
Perform in-house	78 (71)	39 (74)	39 (68)	0.55
Order from lab	32 (29)	14 (26)	18 (32)	

MD, Medical doctor degree; MS, Master of surgery degree; MBBS, Bachelor of medicine and bachelor of surgery undergraduate degrees; INR, Indian rupees; USD, United States dollar; POC, point-of-care.

*Pearson's chi-squared (or Fisher's exact) test for categorical variables comparing practitioners who diagnosed 12 patients or less with TB to those who diagnosed more than 12 patients with TB in the past year; Wilcoxon test for continuous variables.

**Categories are not mutually exclusive.

### Barriers for use of point-of-care tests

**Fig 1:**
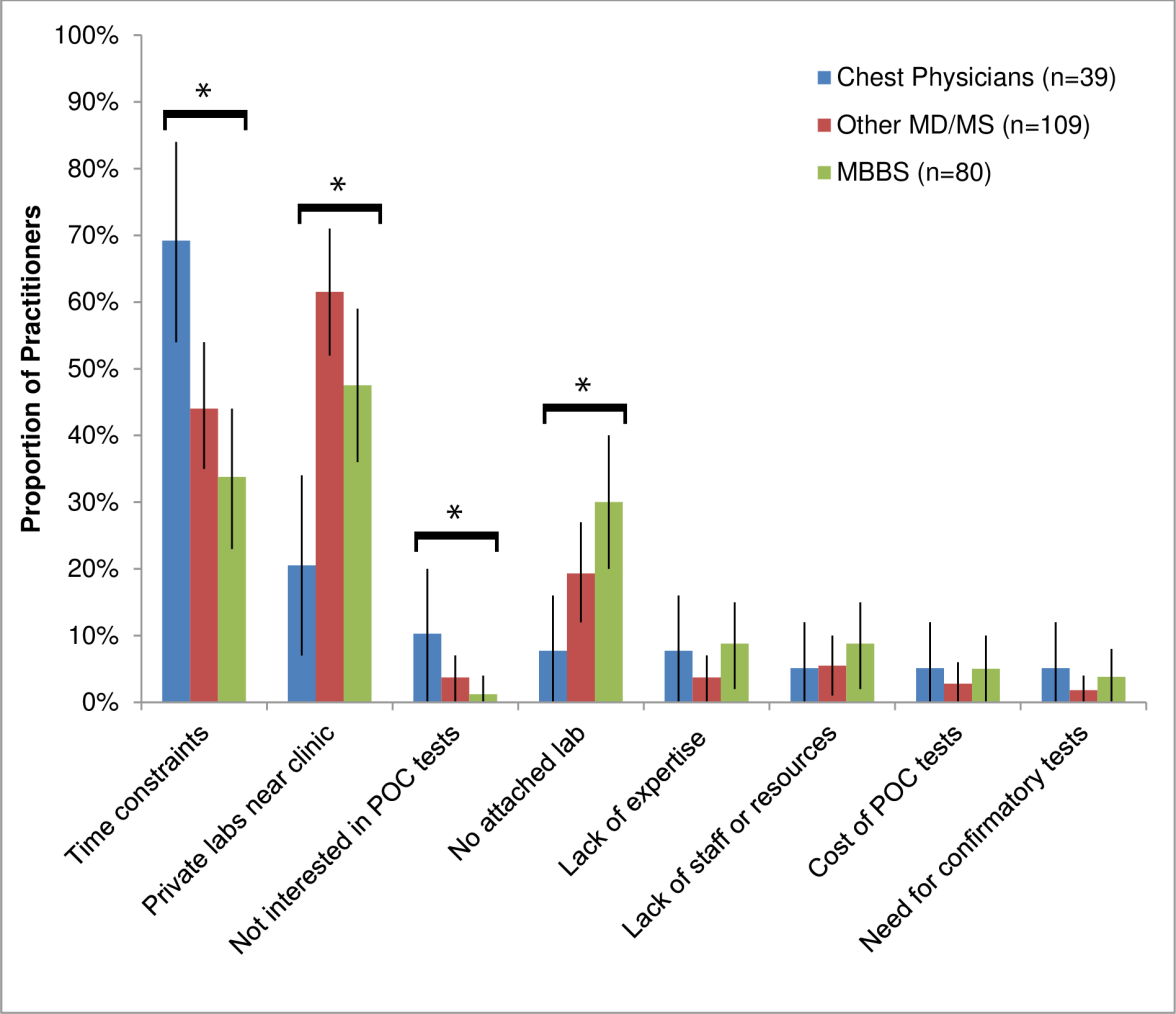
Distribution of challenges in performing point-of-care tests in-house according to practitioners’ level of training (n = 228). Practitioner-reported reasons for not performing rapid point-of-care tests in their health facility among 228 randomly selected private practitioners in Chennai. Practitioners gave multiple responses; thus, response categories are not mutually exclusive. Figures show the distribution of PP-reported challenges in performing POC tests in-house by practitioners’ specialty and level of training. Statistically significant differences across practitioners’ level of training included: time constraints (listed by 69% of chest physicians versus 40% of non-chest specialists, P<0.001), use of nearby private lab services (21% versus 56%, p<0.001), lack of interest in POC tests (10% versus 3%, P = 0.03), and lack of an attached lab (8% versus 24%, p = 0.03). Error bars represent 95% confidence intervals for each estimate. *Indicates statistically significant differences across level of training comparing chest physicians versus non-chest specialists.

When all PPs were asked about major barriers to performing any POC tests in their clinics, chest physicians cited time constraints as their primary barrier (69%, versus 40% of other practitioners, P<0.001), whereas other practitioners also cited the availability of nearby lab services (56%, versus 21% of chest specialists, P<0.001) and the lack of an attached lab (24%, versus 8% of chest specialists, P = 0.03) (Fig 1). There were no significant differences in the PP-reported challenges to using POC tests by patient volume or current POC test usage. Those PPs working in hospitals were substantially less likely to express concern about lack of lab services compared to those in private standalone clinics (39% vs. 57%, P = 0.01), though they also reported greater time management challenges (57% vs. 35%, P<0.01).

### Interest in a hypothetical point-of-care test for TB

Approximately one-tenth (24/228, 11%) of PPs were not interested in any POC testing for TB even for a novel test requiring no equipment that could be completed in 20 minutes, while 38% (87/228) would only order a test for TB from a private lab and 51% (117/228) would perform the novel POC test in-house. Practitioner specialty was independently associated with interest in POC testing in adjusted analyses; chest specialists were half as likely to express interest in performing a novel TB POC test in-house as were other practitioners (aPR 0.5, 95% CI: 0.2–0.9) (Table 3). Practitioners practicing in private hospitals and PPs who did any POC testing were also more likely to opt to use the novel test in-house (aPR 1.3, 95%CI: 1.1–1.7; and aPR 1.8, 95%CI: 1.4–2.4; respectively). Additionally, PPs currently using any POC diagnostic tests were more likely to express interest in performing a novel TB POC test in-house (aPR 1.8, 95%CI: 1.4–2.4). Among those practitioners that currently used point-of-care testing (n = 110), 71% would perform the novel POC test in-house versus 29% would only order a test for TB from a private lab. PPs currently using POC tests and practicing in private hospitals were more likely to favor using the novel test in-house than PPs working in private clinics (43/55, 78% vs. 35/55, 64%, P = 0.09).

**Table 3:** Prevalence and factors associated with interest in performing point-of-care testing for TB in-house among private practitioners in Chennai.

Characteristic*	Interested in POC in-house or on-site (n = 117/228) n/total (%)	Unadjusted PR (95% CI)	P**	Adjusted PR (95% CI)*	P**^‡^
**Gender**					
Male	75/160 (47)	**0.8 (0.6–0.9)**	**0.01**	0.9 (0.8–1.2)	0.61
Female	42/68 (62)	Ref		Ref	
**Years practicing**					
≤20 years	65/117 (56)	1.2 (0.9–1.6)	0.23	1.1 (0.9–1.4)	0.33
>20 years	52/111 (47)	Ref		Ref	
**Practitioner level of training**					
MD (Chest/Pulmonary specialty)	9/39 (23)	**0.4 (0.2–0.8)**	**<0.01**	**0.5 (0.2–0.9)**	**0.03**
MBBS/MS/MD (Non-chest specialty)	108/139 (57)	Ref		Ref	
**Type of facility**					
Private hospital or nursing home	61/99 (62)	**1.4 (1.1–1.8)**	**<0.01**	**1.3 (1.1–1.7)**	**0.01**
Private standalone clinic or polyclinic†	56/129 (43)	Ref		Ref	
**Number of patients diagnosed with TB annually**					
>12 patients	60/118 (51)	1.0 (0.8–1.3)	0.88	1.1 (0.8–1.4)	0.59
≤12 patients	57/110 (52)	Ref		Ref	
**Point-of-care testing practices**					
Any POC diagnostic testing	78/110 (71)	**2.1 (1.6–2.8)**	**<0.001**	**1.8 (1.4–2.4)**	**<0.001**
No POC testing	39/118 (33)	Ref		Ref	
**Usage of sputum smear microscopy for TB**					
Orders at first patient visit for TB diagnosis	54/115 (47)	0.8 (0.6–1.1)	0.23	0.9 (0.7–1.1)	0.35
Not ordered at first patient visit	63/113 (56)	Ref		Ref	
**Knowledge of serological antibody test ban**					
Yes	61/126 (48)	0.9 (0.7–1.2)	0.38	0.9 (0.7–1.2)	0.56
No	56/102 (55)	Ref		Ref	
**Action for pulmonary TB diagnosis**					
Refer to RNTCP or PPM DOTS center	70/131 (53)	1.1 (0.9–1.4)	0.46	1.2 (1.0–1.5)	0.11
Treatment in private sector	47/97 (49)	Ref		Ref	

POC, point-of-care; PR, prevalence ratio.

**P-values were calculated using Poisson regression models with robust standard errors to calculate prevalence ratios.

‡Adjusted for all other variables with data in this column.

†Includes practitioners who practice in government facilities with private practice in the evening.

### Key priorities for use of a point-of-care test for TB

**Fig 2:**
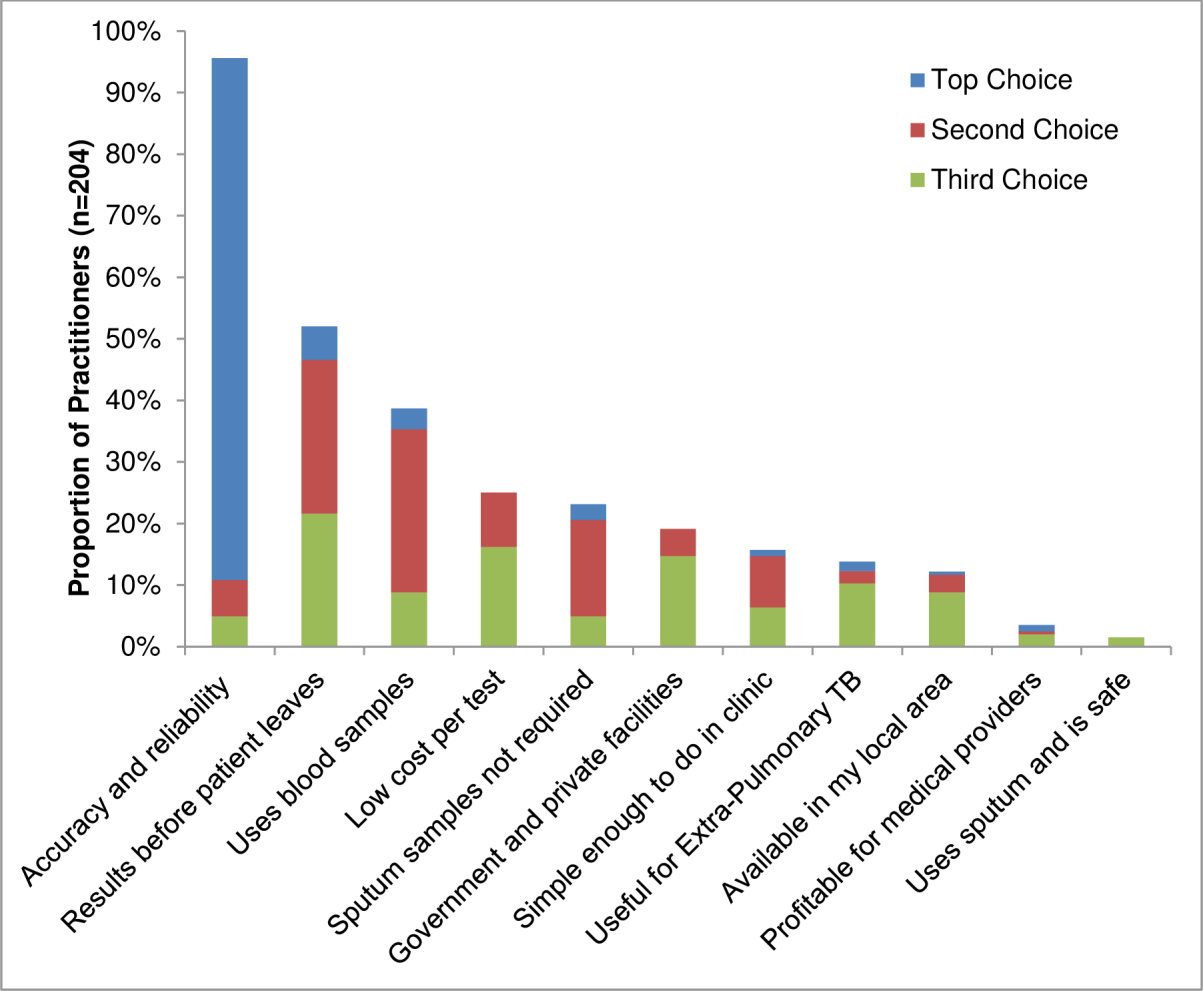
Top three characteristics ranked as priorities by private practitioners for a new point-of-care test for TB in Chennai, India. Private practitioners were asked to rank their top three priorities for a hypothetical new rapid TB diagnostic test under development that could be used to replace the current TB tests and that could potentially be done rapidly in their clinic, like a pregnancy test or blood glucose test. Dark gray bars represent the characteristic ranked as the most important priority by practitioners, light gray presents the second most important characteristic, and medium gray the third most important characteristic. There were 24 practitioners that were not interested in performing POC testing who did not answer this question.

When asked to provide the three most important characteristics of a hypothetical new POC test for TB, 96% (195/204) listed accuracy and reliability as one of the top three priorities (Fig 2). Results available within the first patient encounter was named by 52% (106/204), 39% (79/204) wanted a test that utilized blood samples, 25% (51/204) sought low cost per test, and 23% (47/204) wanted a non-sputum based test. PPs expressed that a maximum cost of 200 INR [IQR 100–500] (approximately US$3.15) for the novel test would be affordable for most patients with symptoms of TB. The top priorities selected among 105 PPs currently performing point-of-care testing were similar, but they valued cost over speed: accuracy and reliability (93%), low cost (50%), use of blood samples (35%), use for extra-pulmonary TB (34%), and results available within the first patient encounter (28%).

## Discussion

If future POC tests are to make an important impact in the fight against TB in high-burden settings like India, they must be implemented in a way that is consistent with current providers’ practices and preferences. This cross-sectional survey of private providers in Chennai provides important insight in this regard. Specifically, only half of practitioners surveyed utilized any POC tests as part of their current diagnostic practices, and utilization was particularly low among chest physicians–who also saw the largest number of patients with TB, were most likely to use private labs, and had the least interest in using a hypothetical new POC test for TB in their clinical practice. These findings suggest that mechanisms to further engage chest physicians, the private lab network, and practitioners who do not currently use POC tests may be needed before any novel POC test for TB could have maximum impact in India.

Our results also suggest the types of POC tests for TB that are likely to be most favorably received and utilized by qualified Indian PPs in urban settings. Overwhelmingly, PPs selected accuracy and reliability as the characteristic most important for a new POC test, suggesting that providers may intrinsically not trust POC tests for TB to be accurate. As in other studies [15], we also found a strong preference for blood rather than sputum as the main sample for TB testing. Rapid test usage was higher among our urban sample of qualified, private practitioners, though still under 50%, than in a study of predominantly rural Indian primary health care providers where only one-quarter used POC tests [16]. These results highlight differences in POC testing practices in different sectors of India’s healthcare system, though willingness to use POC tests for TB was similar in both studies [16]. These findings are consistent with those from rapid diagnostic tests (RDTs) for malaria where provider uptake of these POC tests varied despite widespread availability [17]. Provider reliance on clinical symptoms and lack of confidence in RDT results versus perceived advantages of more familiar tests (i.e., microscopy) posed barriers to clinical usage [18–20] and more than half of providers continue to prescribe antimalarial medications despite a negative RDT result [20,21]. Building on these findings, to achieve successful use and uptake, novel POC diagnostic tests for TB must first show demonstrated accuracy, be usable in a variety of settings, and include efforts to educate private practitioners on test usage and diagnostic capability.

Key challenges to performing POC testing for TB in this study included time constraints, easy access to local private labs and lack of an attached lab facility, such that–when asked about a hypothetical, novel point-of-care test for TB that was accurate, rapid, and equipment-free–only about half of all providers, and less than one-quarter of chest physicians, were interested in using the novel test in-house. In an evaluation of practitioners’ use of current diagnostic tests for TB (i.e., smear, Xpert, culture), nearly one-third of practitioners cited the necessity of sputum samples as the main problem with existing tests [22]. Additionally, the majority of practitioners reported sending patients to private labs for current TB diagnostic testing [22], suggesting potential preferences and challenges that require consideration for optimal implementation of POC TB diagnostic tests. These barriers and recent qualitative research in India [15,23] reinforce the importance of overcoming existing technical, operational, and infrastructure challenges (i.e., time availability, staff capacity, test cost, test specimen, lab space and access) to POC testing to encourage practitioners’ use of future tests [10]. Additionally, the value placed on lab access by practitioners in our study suggests the ideal placement for future POC tests in such community labs to promote use and ensure completion of the test and treat loop for patients with TB.

Although Xpert MTB/RIF has been demonstrated as a feasible point-of-care test in African settings [24], and as a decentralized test in various Indian settings [25,26], it remains a high cost test that requires sophisticated equipment [27]. Low-cost POC tests for HIV and malaria have transformed the management of these diseases, yet the need for similar POC tests for active pulmonary TB remains unmet contributing to delayed diagnoses that fuel the epidemic in high burden countries like India [9,27,28]. The urine lipoarabinomannan (LAM) lateral flow assay represents a simple, low cost diagnostic test that can be used at the point of care, but its niche use is limited to screening for HIV-associated pulmonary TB in patients with advanced immunodeficiency [3,29]. As future POC TB diagnostic tests become available that can be integrated more broadly into outpatient practice, studies such as this one and others [16,23], will be helpful in guiding implementation. Additional research is needed to understand best practices for encouraging use of POC tests among PPs in urban Indian settings.

Several important limitations must be considered in this cross-sectional study. Our sample included only formally trained, qualified practitioners who had diagnosed a case of TB in the past year and also sampled from providers referring to a public-private mix organization in Chennai. This enabled us to attain a high response rate with less recall bias; however, our results may not be representative of all urban private practitioners providing TB care in India. We also used structured questionnaires for data collection to allow us to collect a larger volume of data in the short times available to interview PPs; however, vignette-based questions may be preferred for describing actual practices and identifying priorities for future diagnostics [30]. Additionally, our survey captured practitioner-reported practices and preferences for a hypothetical new TB diagnostic test, but not actual practices (as no such TB test currently exists) or qualitative information on diagnostic priorities or motivations for lack of interest in POC testing for TB. A study using standardized patients to evaluate PP practices has reported a wide gap between what Indian providers know and what they do in real practice [31], which indicates the need for ongoing empiric data collection on practice patterns to better gauge actual POC test usage.

Our findings underscore several important implications for point-of-care testing for TB in urban Indian settings such as Chennai. The low usage and limited interest in POC tests reported by chest physicians in this study represents a potential obstacle to implementation among the providers who see the most patients with TB. Even among practitioners currently using POC tests, nearly one-third preferred to order a test from a private lab even when it could be performed as a rapid POC test on-site. This suggests that certain logistical barriers (e.g., lack of waiting room space) must be overcome, or that a shift in practice patterns may be required, before POC testing for TB will be widely adopted in the private sector in Chennai. Additionally, it will be important to engage small, local laboratories at the community level in urban Indian settings, and take advantage of the strong existing relationships between private doctors and their local laboratories and pharmacies.

## Conclusion

In conclusion, our study demonstrates variable use and interest in point-of-care testing for TB among qualified private practitioners in an urban setting in India. To ensure successful uptake of future POC tests for TB in urban India, such tests may need to be integrated into the private lab system, and chest physicians and providers who do not currently use POC tests may need to be specifically engaged. Accuracy, low physician time demand, low cost, and use of blood samples would all help increase the acceptability of such tests. Ultimately, if we are to use POC diagnostics to improve case detection and halt TB transmission in India, the practices and preferences of private providers with regard to POC testing must continue to be addressed and better understood to ensure test results can be translated into clinical plans for action.
